# What are the drivers of recurrent cholera transmission in Nigeria? Evidence from a scoping review

**DOI:** 10.1186/s12889-020-08521-y

**Published:** 2020-04-03

**Authors:** Kelly Osezele Elimian, Somto Mezue, Anwar Musah, Oyeronke Oyebanji, Ibrahima Soce Fall, Sebastian Yennan, Michel Yao, Patrick Okumu Abok, Nanpring Williams, Lynda Haj Omar, Thieno Balde, Kobina Ampah, Ifeanyi Okudo, Luka Ibrahim, Arisekola Jinadu, Wondimagegnehu Alemu, Clement Peter, Chikwe Ihekweazu

**Affiliations:** 1 Nigeria Centre for Disease Control, Abuja, Nigeria; 2 University of Benin, Benin, Nigeria; 3 University College London, London, UK; 4 World Health Organization, Regional Office for Africa, Brazzaville, Republic of Congo; 5 World Health Organization, Abuja, Nigeria

**Keywords:** Cholera, Scoping review, Drivers, Transmission, Multi-sectoral

## Abstract

**Background:**

The 2018 cholera outbreak in Nigeria affected over half of the states in the country, and was characterised by high attack and case fatality rates. The country continues to record cholera cases and related deaths to date. However, there is a dearth of evidence on context-specific drivers and their operational mechanisms in mediating recurrent cholera transmission in Nigeria. This study therefore aimed to fill this important research gap, with a view to informing the design and implementation of appropriate preventive and control measures.

**Methods:**

Four bibliographic literature sources (CINAHL (Plus with full text), Web of Science, Google Scholar and PubMed), and one journal (African Journals Online) were searched to retrieve documents relating to cholera transmission in Nigeria. Titles and abstracts of the identified documents were screened according to a predefined study protocol. Data extraction and bibliometric analysis of all eligible documents were conducted, which was followed by thematic and systematic analyses.

**Results:**

Forty-five documents met the inclusion criteria and were included in the final analysis. The majority of the documents were peer-reviewed journal articles (89%) and conducted predominantly in the context of cholera epidemics (64%). The narrative analysis indicates that social, biological, environmental and climatic, health systems, and a combination of two or more factors appear to drive cholera transmission in Nigeria. Regarding operational dynamics, a substantial number of the identified drivers appear to be functionally interdependent of each other.

**Conclusion:**

The drivers of recurring cholera transmission in Nigeria are diverse but functionally interdependent; thus, underlining the importance of adopting a multi-sectoral approach for cholera prevention and control.

## Background

Cholera is an acute watery diarrhoeal disease that is caused by the ingestion of food or water contaminated with the toxigenic strains of *Vibrio cholerae* (*V. cholerae*) serogroups O1 or O139 [1]. Cholera is often characterised by a rapid onset of watery diarrhoea, with or without vomiting, and an extensive dehydration [2]. When prompt rehydration therapy is not administered for severe cases, cholera can result in severe clinical sequel including lethargy, unconsciousness, confusion, and a drop in blood pressure and circulatory shock as well as death [3]. The case fatality rate (CFR) from untreated cholera cases can be as high as 30–50%, although the value could be as low as 1% with adequate and prompt care [2].

Modelling exercises indicate that cholera burden remains a global threat, with an estimate of about 2.86 million suspected cases and 95,000 deaths per year [4]. Evidence indicates that these figures could be higher if social, political and economic disincentives for reporting cholera cases are taken into account [5]. Nonetheless, cholera, being an indicator of inequity and social development [6], disproportionately affects low- and middle-income countries. Since the first report of a cholera outbreak in Nigeria in 1970 [7], the country has remained endemic for the disease with several epidemics and high attack rates and CFRs. Moreover, cholera in Nigeria has been increasingly linked with ongoing armed-conflicts, environmental and climatic changes, rapid urbanization and increasing population growth, inadequate emergency or public health responses, traditional and religious beliefs [7–13]. Notably, inadequate access to portable water and poor sanitary conditions remain the principal determinant of cholera transmission in Nigeria, in line with global epidemiology [14].

Adopting a multi-sectoral approach to cholera control and prevention is considered essential in the Global Roadmap strategies, which seek to reduce cholera-related deaths by 90% and eliminate cholera infection in at least 20 out of the 47 endemic countries by 2030 [15]. Following the launch of the Global Roadmap strategies by the Global Task Force on Cholera Control (GTFCC) and partners in 2017, Nigeria has taken strategic steps, ranging from the implementation of oral cholera vaccination campaigns in the northern region of the country to the development of a national strategic action plan [16]. However, a major cholera outbreak across 20 states in Nigeria throughout 2018 was a reminder that the disease remains a serious public health threat. Apart from water, sanitation and hygiene (WASH) and oral cholera vaccination, the 2018 outbreak further underline the need to design and implement complementary public health interventions against recurrent cholera transmission in Nigeria; this will be informed by robust and context-specific evidence on the drivers of cholera transmission in the country. To the best of our knowledge, there is a dearth of evidence in this regard. Two previous reviews on cholera in Nigeria were focused primarily on its epidemiology (causative pathogen, history, geographical distribution, and infection pattern) [17] and description of cholera trends [18], with limited methodological robustness and potential publication bias. For example, these studies provided little or no information about how the literatures were searched and selected, and it was unclear who conducted the searches, selected the studies as well as how the data were extracted, analysed and synthesised. To this end, this study aimed to address the evidence gap in relation to the drivers of recurring cholera transmission in Nigeria and, equally important, to provide a systematic analysis of the operational dynamics of the identified drivers.

## Methods

### Methodological framework

A scoping review was adopted for this study given its flexibility to accommodate diverse study designs and its capacity to address research questions from a relatively broad perspective [19]. Specifically, we adopted the methodological framework as outlined in Arksey and O’Malley [19] guide, which identifies five iterative stages in conducting a scoping review:

- Identifying the research question(s)
- Identifying relevant studies
- Selecting the relevant studies
- Charting the data
- Collating, summarizing and reporting the research findings

Each of these stages are described below.

#### Stage 1: identifying the research question

The overarching research questions that guided this scoping review were: (1) what are the drivers of recurrent cholera transmission in Nigeria, and (2) what is the operational dynamics of the identified drivers relating to cholera transmission in Nigeria?

#### Stage 2: identifying relevant studies

We decided to use a broad definition of search terms and selection of study designs at the outset of this study in order to minimise the chances of missing relevant documents, despite the possibility of generating an enormous number of references during data search. To define the ‘drivers’ of cholera transmission, we adapted the definition proposed by Wepner and Giesecke [20]: developments or factors causing change in or affecting and shaping the transmission of cholera in Nigeria. Additionally, we purposely chose the term ‘transmission’ rather than ‘outbreak or epidemic’ in line with the broad scope of a scoping review.

Four bibliographic literature sources (CINAHL (Plus with full text), Web of Science, Google Scholar and PubMed) and one journal (African Journals Online (AJOL)) were searched to retrieve documents relating to the drivers of cholera transmission in Nigeria (see Supplementary File 1 for details). With the exception of AJOL, the need to seek breadth rather than depth in a scoping review informed the use of the following search terms in all the data sources: “Cholera” OR “Vibrio” OR “Vibrio cholerae” AND “Nigeria”. However, we used the following search terms on AJOL: “Cholera” AND “Nigeria”. Although we initially used a search strategy that included ‘diarrhoea’ and its variants, this was later refined based on early results. Furthermore, we searched other potential document sources including reference lists of selected documents for additional documents that might be useful in addressing the research questions. However, we decided at the outset not to contact experts in the field for ongoing or unpublished works due to the limited time allocated for this research. This stage of the review was undertaken from November 21st, 2018 to November 25, 2018.

#### Stage 3: selection of relevant documents

The following predefined eligibility criteria were used in guiding the inclusion of documents for this scoping review:

- The document had to be a peer-reviewed journal article, conference paper, book chapter, review, case study or short paper
- The document had to focus on cholera, be it in an epidemic or endemic context, in Nigeria
- The document had to include *Vibrio cholerae* or its abbreviation
- The document had to be written in English and published between 1970 (when cholera outbreak was first reported in Nigeria) and the period of data search (November 2018)

We excluded the following documents: editorial, letter, commentary, authored book, book review, or news items as well as documents that focused only on other Vibrio *species* (e.G. *Vibrio parahaemolyticus* and *Vibrio vulnificus*).

Two of the authors (KE and SO) independently applied the predefined eligibility criteria in screening the titles and abstracts of all the selected documents to identify relevance to the research questions. Additionally, the same authors independently screened the full-texts of the selected documents in order to ascertain their eligibility for study; any discrepancies between the two authors were resolved through a discussion or, where an agreement was not reached by consultation with a third author (AM).

#### Stage 4: data charting

Data charting or extraction describes the synthesis and interpretation of data through sifting and sorting in accordance with the common themes [21]. Thus, we developed a comprehensive provisional data extraction form using an inclusive approach, which was then reviewed by all the authors to ensure the variables were relevant. Comments and suggestions from co-authors were addressed to develop the final data extraction form (Supplementary File 2). For data entry and management, however, the data extraction form was translated from MS Word into Excel format. Two authors (KE and SO) conducted data extraction process while discrepancies were addressed using the approach outlined in the previous stage.

#### Stage 5: collating, summarising and reporting the results

Two sets of narrative accounts of findings were presented. The first account focused on a bibliometric analysis of the documents, with a view to understanding the nature and general information about the identified documents (e.g. study design, clinical features, location studies were undertaken etc.). The second account involved organising the documents thematically according to the identified drivers. Using Stata version 15 (StataCorp LP, College Station, TX, USA), findings of the bibliometric analysis were presented using descriptive statistics which included frequencies and percentages for binary and categorical variables, and mean (standard deviation) and median (inter-quartile range) for continuous variables with normal and non-normal distributions, respectively. Furthermore, a systematic analysis was conducted to provide an in-depth understanding of the operational dynamics (mechanism of actions) of the identified drivers. Findings from this analysis were presented using Vensim software (version PLE × 32). As the development of reporting guidance for the conduct and reporting of scoping reviews is underway, the Preferred Reporting Items for Systematic Reviews and Meta-Analyses (PRISMA) protocols were followed in reporting our findings where appropriate [22] (see Supplementary File).

## Results

### Description of documents

**Fig. 1:**
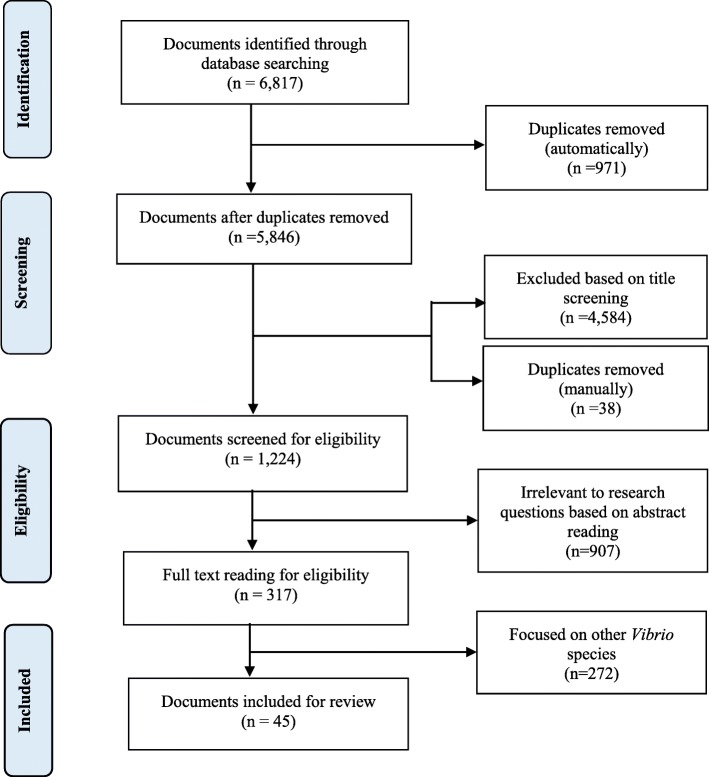
A flowchart showing the selection of documents for the scoping review

**Fig. 2:**
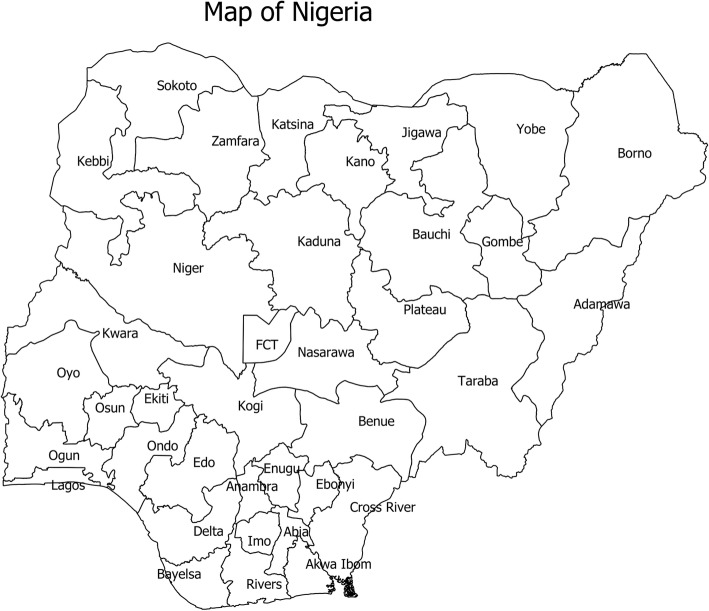
A map of Nigeria showing the 36 states and Federal Capital Territory. Source: Risk Communication Unit of the Nigeria Centre for Disease Control; developed using ArcGIS software version 10.7

Of the 317 documents screened for eligibility, 45 documents met the predefined inclusion criteria and formed the basis for this study (Fig. 1). The characteristics of these documents are summarised in Table 1. The majority of the documents were peer-reviewed journals (88.89%), over half (64.44%) were published between 2011 and 2018 while only four (8.89%) documents were published prior to 1990. The majority (64.44%) of the studies were undertaken in the context of cholera epidemics, and predominantly cross-sectional (62.22%) in terms of epidemiological design. Nearly half (48.89%) of the documents reported an average case fatality rate of 6.53%. Regarding microbiological investigations, 48.49 and 11.11% of the documents conducted culture and rapid diagnostic test, respectively. Although a substantial number of documents were not specific regarding *V. cholerae* biotype, El-Tor (8.89%) and a combination of Classical and El-Tor (4.44%) biotypes were reported. Moreover, there were more reports of Ogawa (17.78%) than Inaba (2.22%) serotype, although a combination of both serotypes (2.22%) was also reported. Fig. 2 shows the 36 states and the Federal Capital Territory in Nigeria.

**Table 1:** Baseline characteristics of reviewed documents (*N* = 45)

**Characteristic**	**Frequency (%)**
**General characteristics**	
**Authors’ affiliation**	
Academic institution	22 (48.89)
Academic and government	7 (15.56)
Academic and hospital	1 (2.22)
Academic and International NGO	1 (2.22)
Governmental health institution	12 (26.67)
Governmental health institution and International NGO	1 (2.22)
International NGO	1 (2.22)
**Document type**	
Conference proceeding	5 (11.11)
Peer-reviewed journal	40 (88.89)
**Publication period**	
< 1990	4 (8.89)
1990–2000	6 (13.33)
2001–2010	6 (13.33)
2011–2018	29 (64.44)
**State where study was undertaken**	
Akwa-Ibom and Cross-River	1 (2.22)
Bauchi	1 (2.22)
Bauchi and Gombe	1 (2.22)
Bauchi, Borno and Gombe	2 (4.44)
Bauchi, Borno and Osun	1 (2.22)
Benue	1 (2.22)
Borno	2 (4.44)
Cross-River	4 (8.89)
Jigawa	2 (4.44)
Kaduna	4 (8.89)
Kano	2 (4.44)
Katsina	1 (2.22)
Lagos	2 (4.44)
Nasarawa	1 (2.22)
Niger	1 (2.22)
Ogun	1 (2.22)
Osun	2 (4.44)
Oyo	7 (15.56)
Rivers	1 (2.22)
Multiple states (> 3 states)	8 (17.78)
**Study context**	
Epidemic	29 (64.44)
Endemic	9 (20.00)
Endemic and epidemic	6 (13.33)
Unspecified	1 (2.22)
**Study approach**	
Prospective	18 (40.00)
Retrospective	23 (51.11)
Prospective and retrospective	3 (6.67)
Unclear	1 (2.22)
**Study design**	
Case-control	10 (22.22)
Cross-sectional	28 (62.22)
Review	4 (8.89)
Unspecified	3 (6.67)
**Median sample size reported in documents (IQR)**^a^	329 (109–1220)
**Age group of study participants**	
All age groups	17 (37.78)
Adults	2 (4.44)
Children under-5 years	1 (2.22)
Children under-14 years	1 (2.22)
Unspecified	24 (53.33)
**Funding for study**	
Unspecified	41 (91.11)
Yes	4 (8.89)
**Ethical approval for the study**	
Unspecified	34 (75.56)
Yes	11 (24.44)
**Clinical characteristics**	
**Data collection approach**	
Record extraction	4 (8.89)
Microbiological examination	7 (15.56)
Questionnaire	9 (20.00)
Record extraction and microbiological examination	1 (2.22)
Questionnaire and microbiological examination	9 (20.00)
Record extraction and questionnaire	2 (4.44)
Record extraction, questionnaire and microbiological examination	1 (2.22)
Record extraction, questionnaire and observation	2 (4.44)
Unspecified	10 (22.22)
**Report of case fatality rate (%)**	
No	23 (51.11)
Yes	22 (48.89)
**Mean (SD) case fatality rate**^b^	6.53 (3.90)
**Report of attack rate**	
No	40 (88.89)
Yes	5 (11.11)
**Location of sample collection**	
Community	11 (24.44)
IDP camp	1 (2.22)
Primary	1 (2.22)
Secondary	1 (2.22)
Tertiary	4 (8.89)
Primary and secondary	1 (2.22)
Secondary and tertiary	3 (6.67)
Tertiary and private	1 (2.22)
Unspecified health facility	2 (4.44)
Unspecified health facility and community	4 (8.89)
Unspecified health facility and IDP camp	1 (2.22)
Unspecified	15 (33.33)
**Culture**	
No	11 (24.44)
Yes	22 (48.89)
Unspecified	12 (26.67)
**Rapid diagnostic test performed**	
No	40 (88.89)
Yes	5 (11.11)
**Identified biotype**	
Classical	1 (2.22)
El-Tor	4 (8.89)
Classical & El-Tor	2 (4.44)
Atypical El-Tor	1 (2.22)
Unspecified	37 (82.22)
**Identified serogroup**	
O1	13 (28.89)
Non-O1	2 (4.44)
Unspecified	30 (66.67)
**Identified serotype**	
Ogawa	8 (17.78)
Inaba	1 (2.22)
Ogawa and Inaba	1 (2.22)
Unspecified	35 (77.78)

^a^ Based on 34 out of 45 documents

^b^Based on 22 out of 45 documents

### Thematic analysis of documents

Five broad factors were identified as potential drivers for recurring cholera transmission in Nigeria: (1) social, (2) biological, (3) environmental and climatic, (4) health systems, and (5) multiple (a combination of two or more factors) drivers (Table 2). Over three-quarters of the documents were related to social drivers, making it the most frequently reported driver of cholera transmission. We identified ‘multiple’ factors as the second driver (i.e. a combination of two or more factors) while biological drivers were the least reported.

**Table 2:** Distribution of the drivers of cholera transmission in Nigeria, *N* = 45

**Driver**	**Citation frequency of the reviewed documents**
Social (demographic, cultural and economic)	35
Biological (host and genetics)	3
Environmental and climatic	11
Health systems-related	8
Multiple^a^	27

^a^Climatic and social drivers (*n* = 2); social and biological drivers (*n* = 3); social and health systems-related drivers (*n* = 1); and two or more drivers (*n* = 21)

Table 3 describes the identified drivers of cholera transmission in Nigeria in detail, while citing specific examples from the reviewed documents.

**Table 3:** A description of the drivers of cholera transmission in Nigeria

**Cholera transmission driver**	**Level/category**	**Examples from the reviewed documents**
Social	Micro-level	
	• Household	• Large household size and over-crowdedness • Poor sanitation and hygiene practices • Poor sewage disposal practices • Socioeconomic status (income and/or education) • Inter-family transmission/contact • Reliance on contaminated water sources (e.g. open wells)
	Micro-level	
	• Individual	• Open defecation • Consumption of seafood, sea and estuarine waters • Inadequate knowledge, and poor attitude and practices towards cholera • Religious beliefs (e.g. reluctance among female patients to seek care from male-dominated health providers) • Superstitious beliefs and/or myths
	Macro-level	
	• Governance/political	• Water scarcity due to inadequate power supply (electricity) • Inadequate public water supply
	Macro-level	
	• Trade and migration	• Increased fishing activities (e.g. trade traffic on the Calabar river estuary) • Increased migration and internal displacement of people (primarily due to armed conflicts)
Biological	Genetics	• Acquisition of resistance genes • Changes in the major virulence determinant genes
Environmental and climatic	Environmental	
	• Natural disaster	• Flooding
	Environmental	
	• Human-made	• Contaminated water sources by poor sewage disposal, waste dumps, abattoir, among others. • Street-vended and sachet water
	Climatic	• Unfavourable weather variables including rainfall and temperature
Health systems-related	Health provision	• Inadequate funding for surveillance system • Inadequate training of health workers and health facilities • Inadequate supply of essential materials including oral cholera vaccine and oral rehydration solutions • Limited capacity for prompt and accurate cholera diagnosis, and delays in the notification of cholera cases
	Health seeking	• Delay in seeking care at formal health facilities after cholera onset • Inadequate knowledge, attitude and practices towards cholera •
	Interphase between health provision and seeking	• Lack of trust by community members for formal health systems • Religious and/or superstitious beliefs
Multiple	A combination of two or more drivers	• Over-crowdedness due to increasing population and natural disasters and human-made factors (e.g. conflicts) • Fragile surveillance system and limited political-will

#### Social drivers of cholera transmission

Social drivers of cholera transmission appear to be operating at two levels: micro and macro. At the micro-level, social drivers are either operating at the household- or individual-level. However, the majority of social drivers at the household-level seem to drive cholera transmission indirectly through the enforcement of other drivers. For instance, a large household size drives cholera transmission indirectly by creating an over-crowded environment, thereby enhancing the likelihood of a person-person transmission of cholera via a common source, such as contaminated water or food. It is worth noting that social drivers operating at the individual-level tend to be behavioral– or cultural–related, such as open defecation. Social drivers operating at the macro-level appear to drive cholera transmission through governmental or political actions and inactions as opposed to individual and household drivers. Although driving cholera transmission indirectly, these social drivers have the capacity to cause a widespread cholera outbreak. Availability of portable water is fundamental to cholera prevention and control, but is often dependent on constant power supply for functionality. Moreover, trade and migration exert their influence on cholera transmission in a similar manner; and armed-conflicts and terrorism, especially that which is perpetuated by Boko Haram terrorist group in the north-east region of the country, drives cholera transmission indirectly by creating enabling conditions, such as including over-crowdedness, disruption of clean water, exacerbation of malnutrition, and among many others.

#### Biological drivers of cholera transmission

Biologically, recurrent cholera transmission appears to be driven by genetic mutation and the resulting acquisition of resistant genes and changes in major virulence determinant genes by *V. cholerae*. In addition, the role of biological drivers appears to be dependent, partly, on the activities of certain social drivers operating at the individual level (e.g. poor attitude towards cholera infection and treatment).

#### Environmental and climatic drivers of cholera transmission

Environmental drivers pertain to natural disasters, such as flooding, or human-made events, such as water source contamination. Natural disasters appear to operate at the macro-level and drive cholera transmission indirectly by creating an enabling environment for *V. cholera* proliferation or, by enforcing other drivers. In contrast, environmental drivers of human-made events or anthropogenic activities tend to drive cholera transmission directly by serving as a reservoir or a transmission media (e.g. open-wells) for the causative organism *V. cholerae*. With respect to climatic drivers, rainfall and temperature are the predominant factors and seem to drive cholera transmission indirectly; for instance, through the genetic mutation of *V. cholerae* due to changing environmental levels of temperature).

#### Health systems-related drivers of cholera transmission

Health systems-related drivers were found to be operating primarily in three areas: health provision by health professionals, health seeking by community members, and interphase between health provision and health seeking. Essential to health provision is a relatively fragile surveillance system that is enforced by inadequate funding and training of health professionals as well as limited capacity for cholera diagnosis and case notifications. In contrast, health seeking within the community tends to drive cholera transmission largely due to individual characteristics including inadequate knowledge and inappropriate attitude and practices towards cholera. Interestingly, although implicitly stated in the relevant documents, certain health-related drivers of cholera transmission in Nigeria operate at the interphase between healthcare provision and healthcare seeking. An example depicting this scenario is the lack of community trust for a surveillance system and formal health care facilities, which could be instigated by poor attitudes of health workers to patients.

#### Multiple drivers of cholera transmission

Apart from social drivers with the highest frequency of citations, most of the reviewed documents are centred upon at least two drivers (multiple drivers) of recurring cholera transmission concurrently. For instance, over-crowding which increases the risk of cholera transmission was found to result from increased influx of a displaced population into a community due to either a natural disaster (e.g. flooding) or armed conflict (i.e. a social driver operating at the macro-level).

#### Dynamics of the drivers of cholera transmission in Nigeria

**Fig. 3:**
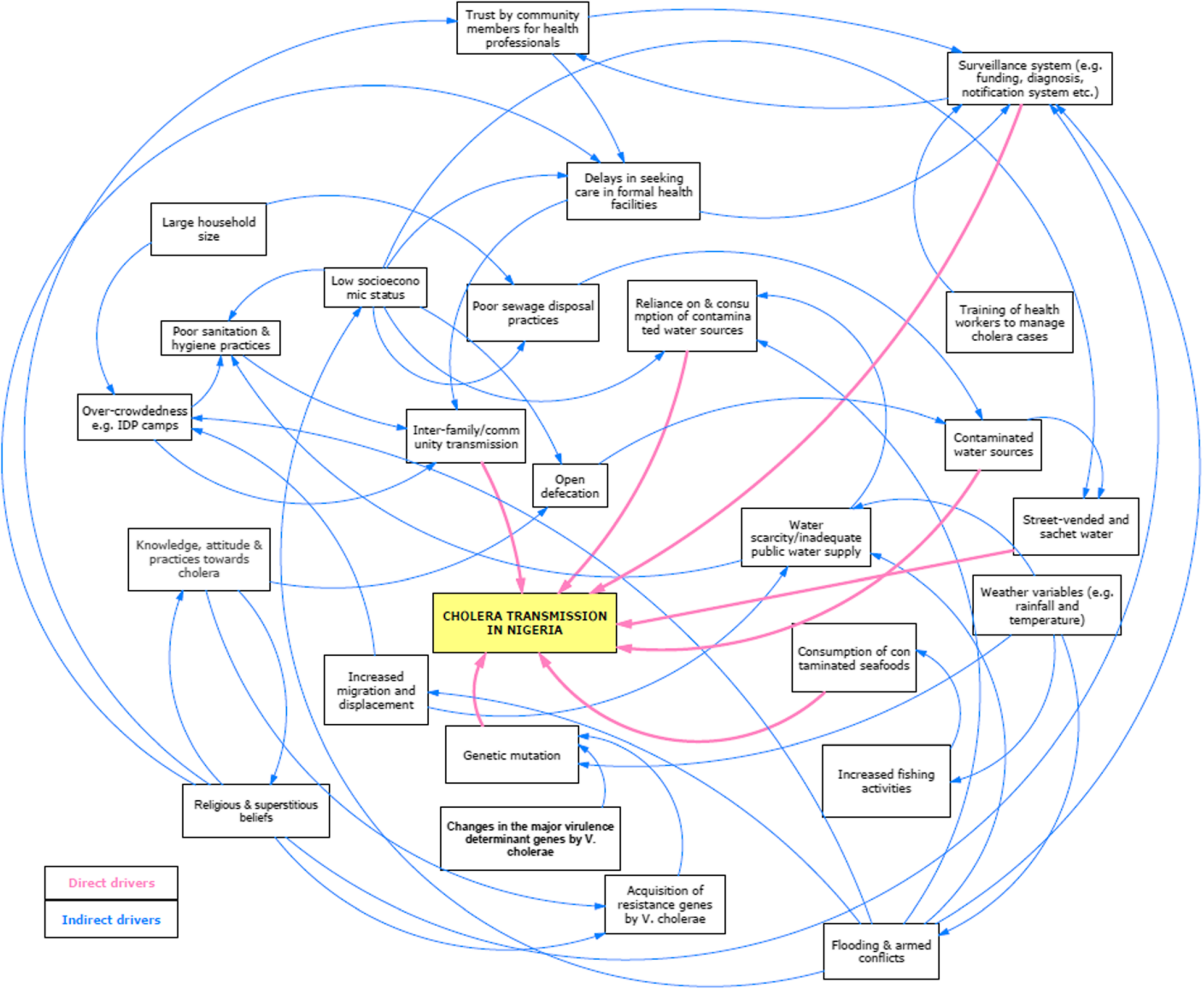
The dynamics of cholera drivers in Nigeria

Figure 3 below depicts the operational dynamics of the identified drivers of cholera transmission in the Nigerian context. The majority of the drivers of cholera transmission are interdependent of each other, such that they are either enforcing the activities of other drivers or their activities are being enforced by others to bring about cholera transmission. For instance, religious and superstitious beliefs (lower left of Fig. 3) could influence or be influenced by community knowledge, attitude and practices towards cholera, as well as community trust for health system and delay in seeking health care following symptom onset. Overall, the majority of the drivers of recurrent transmission of cholera in Nigeria seem to be intertwined rather than operating in isolation.

## Discussion

### Summary of key findings

The findings from this scoping review are timely given the global urgency to address recurring cholera outbreaks, especially in countries with high attack and case fatality rates. Overall, this study has identified the drivers of recurrent cholera transmission in Nigeria. The identified drivers pertained to a combination of social, biological, environmental and climatic, and health systems-related factors.

### Interpretation of key findings

A majority of the reviewed documents [7, 13, 17, 23–50] pertained to social drivers. Notably, the significant impact of cultural and behavioural factors on individual social drivers of cholera transmission (e.g. open defecation, food consumption among others) was noted, as well as the prominence of religious, superstitious and traditional beliefs in Nigeria [51, 52]. This study has therefore underlined the need to address erroneous socio-cultural beliefs, especially in rural areas, in ongoing efforts to mitigate cholera transmission. Open defecation is a prevalent practice in many areas of Nigeria, as indicated by a recent ranking in which the country was ranked third after India and China [53]. Individual-based approach to reverse this harmful practice in Nigeria has been demonstrated to be ineffective [54], thus necessitating the importance of adopting a multi-sectoral approach to designing preventive measures against this harmful practice. The potential impact of increasing armed-conflicts on recurrent cholera transmission is not unique to Nigeria alone but is a universal phenomenon [17], as evidenced by studies in Yemen [55] and Kenya [56]. Although conflict resolution is not the mandates of national public health institutions, such as the Nigeria Centre for Disease Control, findings from this study indicate that interactions and collaborations among the public health, security, community and religious sectors are imperative to addressing the impact of armed-conflict on recurrent cholera transmission.

The mechanism by which biological factors drive cholera transmission appears to be largely genetic-based, as opined by Adewale et al. [57], Marin et al. [58], and Oyedeji et al. [42]. Generally, genetic mutation has been demonstrated to be linked with the emergence of new, virulent and drug-resistant strains of *V. cholerae* [3]. For example, Hu et al. [59] argue that the seventh cholera pandemic became prominent in 1961 after *V. cholerae* underwent series of mutations, with suitable niches in the Middle East and gene sources from Makassar to aid the genetic events. This hypothesis is supported by Marin et al. [58] wherein the 2009 and 2010 cholera outbreaks in Nigeria were linked to multidrug resistant atypical El Tor strains. Further supporting this evidence are findings in India [60] and Mozambique [61]. In practice, continuous surveillance of antibiotic resistance by public health institutions will be critical for mitigating cholera transmission in Nigeria.

Rainfall [7, 26, 30, 32, 45, 62] and temperature [7, 26, 50] were identified as primary climatic drivers of cholera transmission in Nigeria. The association between cholera outbreaks and climatic drivers, particularly seasonal tropical rainfall, is well documented in other contexts [63–66]. Two mechanistic models for cholera transmission with respect to rainfall have been proposed: cholera transmission tends to be enhanced given the high tendency for consumption of contaminated water and worsening sanitary conditions from floods; and the ease with which water becomes contaminated by freshly excreted bacteria resulting from washout of open-air defecation sites, or overflows from pit latrines during and after rainfall. These models have been validated in South Sudan [67] and Senegal [68]. A 2-fold increase in cholera cases with a 1 °C increase in temperature at 4 months lag has been reported in Zanzibar [69], indicating the importance of temperature in cholera transmission. Flooding was also identified as an environmental driver of cholera transmission in Nigeria [41, 70]. Flooding increases cholera transmission by (1) disrupting access to or contaminating safe water sources; (2) affecting sanitation conditions; and (3) limiting access to essential health services [71–73]. Although we did not identify studies that specifically explored the association between droughts and increased cholera transmission in Nigeria, evidence [74] suggests that the two variables are significantly linked.

Eight studies [12, 17, 24, 32, 41, 50, 70, 75] found the potential role of health systems-related factors in driving the transmission of cholera in Nigeria. Regarding health service delivery, evidence centred on inadequate and inefficient surveillance system, as well as inadequate laboratory diagnostic capacity; in addition, poor technical capacity of health workers to manage cholera cases, especially in rural areas. In rural areas in Nigeria, it is not surprising to encounter health workers with inadequate training on cholera case management, as well as with inadequate supply of emergency response kits [12]. From the perspective of health care seeking by community members, religious and traditional beliefs play a significant role in driving cholera transmission. The effect of erroneous beliefs—e.g. cholera is caused by an evil spirit (or apparition) or “it’s Gods’ will”—on cholera transmission however appears to be indirect by hindering health care seeking following cholera infection. Such beliefs have also been linked with poor outcomes of control and prevention interventions in endemic areas of West Africa [13]. This evidence further reiterates the need for actively engaging both community and religious stakeholders in the fight against cholera in Nigeria and, perhaps, in other endemic countries with similar socio-cultural profile.

Twenty-seven documents captured multiple drivers of cholera transmission in Nigeria, underlining the inter-dependency of the identified drivers and a need for an ‘all-inclusive’ or a multi-sectoral control and prevention strategy. This approach is being advocated by the Global Task Force on Cholera Control (GTFCC) and partners for cholera control worldwide [15]. Importantly, this evidence suggests that improving access to WASH services and provision of oral cholera vaccines alone might not be sufficient to yield the desired goals, as stipulated in the GTFCC Global Roadmap strategic goals. That is, there should be a balance between investment in preventive and control measures and the recipient community in order to ensure adherence and wider uptake during a cholera outbreak [76].

### Strengths and limitations

This study has the advantage of adopting a robust methodological framework with a number of shared processes with systematic reviews, and less prone to publication bias given the emphasis on breadth (covering all available material) over depth (providing a detailed analysis and appraisal of a smaller number of studies) [19]. However, some limitations associated with this study are worth acknowledging. Firstly, unlike systematic reviews, the quality of evidence in the reviewed documents was not formally appraised. Although the primary focus of a scoping review is addressing study specific questions rather than quality assessment [19], the preponderance of cross-sectional studies, with limited capacity to establish causality, needs to be considered in interpretation of our findings. Secondly, the adopted framework for a scoping review does not allow for synthesis, suggesting that only narrative or descriptive account of evidence could be achieved. However, we conducted an in-depth analysis of the operational dynamics of the identified drivers. An optional stage in the adopted framework for a scoping review is a ‘consultation exercise’ whereby the views of practitioners and experts in a field are sought in order to provide additional references about the subject of discussion that the review alone would not have identified. In addition to resource implications of implementing this step, the current research team members, many of whom are experts in the field, however provided inputs in synthesising the operational dynamics of cholera drivers.

## Conclusion

These findings from this scoping review indicate that the drivers of recurring cholera transmission in Nigeria are diverse but functionally interdependent, underlining the importance of a multi-sectoral approach towards cholera prevention and control.

## Supplementary information

**Additional file 1.**

**Additional file 2.**

**Additional file 3.**

## Data Availability

The documents used for this study are available upon request to the corresponding author.

## Abbreviations

PRISMA: The Preferred Reporting Items for Systematic Reviews and Meta-Analyses
WASH: Water, Sanitation and Hygiene
