# Supplementary material for: What are the drivers of recurrent cholera transmission in Nigeria? Evidence from a scoping review
Source: BMC Public Health. 2020 Apr 3;20:432. doi: 10.1186/s12889-020-08521-y (PMC7118857; doi:10.1186/s12889-020-08521-y)
Supplement: Supplementary file 1 — Additional file 1. [file 12889_2020_8521_MOESM1_ESM.docx]

**Supplementary File 1**

| **Document sources and search outcomes** | |
| --- | --- |
| **Databases** | **Number of hits** |
| CINAHL Plus with full text | 1,268 |
| Web of Science | 4,464 |
| Google Scholar [search terms restricted to ‘all in title’] | 94 |
| PubMed | 114 |
| African Journals Online (AJOL)* | 877 |
| **Total number of documents** | **6,817** |
| Search terms: Cholera OR Vibrio OR Vibrio cholerae AND Nigeria  *Search terms used were: cholera AND Nigeria | |
