# Supplementary material for: What are the drivers of recurrent cholera transmission in Nigeria? Evidence from a scoping review
Source: BMC Public Health. 2020 Apr 3;20:432. doi: 10.1186/s12889-020-08521-y (PMC7118857; doi:10.1186/s12889-020-08521-y)
Supplement: Supplementary file 2 — Additional file 2. [file 12889_2020_8521_MOESM2_ESM.docx]

**Updated data extraction dictionary**

| **Variable** | **Required information** |
| --- | --- |
| Doc ID | Continuous numbers |
| Initials of data extractor |  |
| First author (only the last name) |  |
| Year of publication |  |
| Affiliation of ALL the authors | -Academic institutions only  -INGO/NGOs (only)  -Governmental agencies  -Hospital (only)  -Academic and other institutions  -Not Reported (NR) |
| Other authors’ country | Multiple answers are permitted |
| Study title |  |
| Study objective |  |
| Document type | -Journal article  -Review  -Book chapter  -Conference proceeding  -Add as you see fit |
| Study approach | -Prospective  -Retrospective |
| Study design | -Observational (cross-sectional, case-control or cohort study)  -Review  -Intervention (randomized control study or cluster randomized control study)  -Qualitative interviews (key informant interview and/or focus group discussion)  -NR (e.g. book chapters) |
| Study state | Not Applicable for a review |
| Study LGA | Not Applicable for a review |
| Context of cholera transmission | -Outbreak (epidemic)  -Normal transmission (endemic)  -Unspecified |
| Year study/outbreak started | Include the month if stated |
| Year study/outbreak ended | Include the month if stated |
| Year outbreak peaked | Include the month if stated |
| Sample population studied | -Community members  -Hospital patients  -Internally-displaced persons  -Mixed  -NR |
| Sample size studied |  |
| Sampling method | -Probability sampling*  -Non-probability sampling** |
| Method used for data collection | -Face-to-face household/site survey  -Telephone interview  -Chart/record extraction  -NA |
| Sample collection location | -Cholera treatment centre (usually within a health facility)  -Primary health centre  -Secondary/tertiary hospital  -IDP camp  -Schools  -NGO facility  -Community (population-based)  -NR (did not specify location) |
| Specimen source/mode of injection | -Clinical  -Environmental (including water)  -Food  -Mixed (two or more sources) |
| Criteria for cholera definition | Also include the guideline if reported |
| Molecular characterisation of specimen | Yes  No |
| V. cholerae serogroup | 01  0139 |
| V. cholerae biotype | Classical  El Tor |
| V. cholera serotype | Inaba  Ogawa  Hikojima |
| Outbreak origin (only for outbreak cases) | Environmental  Clinical |
| Age group of study population | -Pregnant and breastfeeding  -Infant and child (<2years)  -Children (2-9 years)  -Children and Adolescents (10-18 years)  -Adults (>years)  -All age group |
| Proportion (%) of male population | Calculate by subtracting from the total number of sample size; inference on the female proportion will be made later |
| Attack rate |  |
| Case fatality rate |  |
| Clinical features of affected population | Report only the most serious and predominant features |
| Broad drivers of cholera reported | -Environmental factors  -Host factors  -System factors |
| Specific environmental drivers 1 |  |
| Specific environmental driver 2 |  |
|  |  |
| Specific environmental driver 3 |  |
| Specific environmental driver 4 |  |
| Specific environmental driver 5 |  |
| Specific host driver 1 |  |
| Specific host driver 2 |  |
| Specific host driver 3 |  |
| Specific host driver 4 |  |
| Specific host driver 5 |  |
| Specific system driver 1 |  |
| Specific system driver 2 |  |
| Specific system driver 3 |  |
| Specific system driver 4 |  |
| Specific system driver 5 |  |
| Limitations of study identified by authors | Typically in the discussion |
| Limitations identified by reviewers | Overall self-assessment e.g. was standard definition of cholera used? |
| Funding | Yes  No  Unspecified |
| Funding source (if answer to previous question is ‘Yes’ | -Governmental  -Private/industry  -INGO/NGO  -No funding  -Not applicable (book chapter) |
| Ethical approval | Yes  No  Not mentioned |
